# An Overview of Cancer in the First 315,000 *All of Us* Participants

**DOI:** 10.1371/journal.pone.0272522

**Published:** 2022-09-01

**Authors:** Briseis Aschebrook-Kilfoy, Paul Zakin, Andrew Craver, Sameep Shah, Muhammad G. Kibriya, Elizabeth Stepniak, Andrea Ramirez, Cheryl Clark, Elizabeth Cohn, Lucila Ohno-Machado, Mine Cicek, Eric Boerwinkle, Sheri D. Schully, Stephen Mockrin, Kelly Gebo, Kelsey Mayo, Francis Ratsimbazafy, Alan Sanders, Raj C. Shah, Maria Argos, Joyce Ho, Karen Kim, Martha Daviglus, Philip Greenland, Habibul Ahsan, On behalf of the All of Us Research Program Investigators

**Affiliations:** 1 Department of Public Health Sciences, University of Chicago, Chicago, Illinois, United States of America; 2 Institute for Population and Precision Health, University of Chicago, Chicago, Illinois, United States of America; 3 Comprehensive Cancer Center, University of Chicago, Chicago, Illinois, United States of America; 4 Vanderbilt University Medical Center, Nashville, Tennessee, United States of America; 5 Brigham and Women’s Hospital, Boston, Massachusetts, United States of America; 6 Hunter College City University of New York, New York, New York, United States of America; 7 University of California San Diego Health, La Jolla, California, United States of America; 8 Mayo Clinic, Rochester, Minnesota, United States of America; 9 The University of Texas Health Science Center at Houston, Houston, Texas, United States of America; 10 National Institutes of Health, Bethesda, Maryland, United States of America; 11 National Institutes of Health, Leidos, Inc, Frederick, Maryland, United States of America; 12 Johns Hopkins University School of Medicine, Bethesda, Maryland, United States of America; 13 Northshore University Health System, Evanston, Illinois, United States of America; 14 Rush Alzheimer’s Disease Center, Rush University Medical Center, Chicago, Illinois, United States of America; 15 Division of Epidemiology and Biostatistics, School of Public Health, University of Illinois at Chicago, Chicago, Illinois, United States of America; 16 Department of Preventive Medicine, Northwestern University Feinberg School of Medicine, Chicago, Illinois, United States of America; 17 Department of Medicine, University of Chicago, Chicago, Illinois, United States of America; 18 Institute for Minority Health Research, College of Medicine, University of Illinois at Chicago, Chicago, Illinois, United States of America; CNR, ITALY

## Abstract

**Introduction:**

The NIH *All of Us* Research Program will have the scale and scope to enable research for a wide range of diseases, including cancer. The program’s focus on diversity and inclusion promises a better understanding of the unequal burden of cancer. Preliminary cancer ascertainment in the *All of Us* cohort from two data sources (self-reported versus electronic health records (EHR)) is considered.

**Materials and methods:**

This work was performed on data collected from the *All of Us* Research Program’s 315,297 enrolled participants to date using the Researcher Workbench, where approved researchers can access and analyze *All of Us* data on cancer and other diseases. Cancer case ascertainment was performed using data from EHR and self-reported surveys across key factors. Distribution of cancer types and concordance of data sources by cancer site and demographics is analyzed.

**Results and discussion:**

Data collected from 315,297 participants resulted in 13,298 cancer cases detected in the survey (in 89,261 participants), 23,520 cancer cases detected in the EHR (in 203,813 participants), and 7,123 cancer cases detected across both sources (in 62,497 participants). Key differences in survey completion by race/ethnicity impacted the makeup of cohorts when compared to cancer in the EHR and national NCI SEER data.

**Conclusions:**

This study provides key insight into cancer detection in the *All of Us* Research Program and points to the existing strengths and limitations of *All of Us* as a platform for cancer research now and in the future.

## Introduction

Cancer was the leading cause of death in the United States after cardiovascular diseases in 2020, with over 600,000 cancer-related deaths and a further 1.8 million expected diagnoses [1–5]. Although treatments are improving and personalized medicine promises advancements, cancer diagnoses are expected to increase substantially over the next decade, due mainly to the aging population in the US and modifiable behavioral/lifestyle factors [1,2,6]. The risk of developing cancer depends on the complex interplay of factors including genes, age, and gender, lifestyle and behavioral factors such as diet, energy balance, physical activity, tobacco and alcohol use; endogenous factors such as hormones and growth factors; medication and drug use; infectious agents; and environmental exposures [1,6]. Precision medicine and precision health, which consider the patient as an individual, hold promise for cancer research [7–10]. For instance, individuals with similar diagnoses often receive the same treatment despite observations that efficacy varies by patient. Additionally, new approaches to precision *prevention* and early detection, informed by an enriched understanding of the etiology and natural history of cancer, could improve clinical interventions.

With over one million participants, the *All of Us* Research Program will have the scale to enable research on myriad diseases, especially cancer [11–13]. The program’s focus on diversity and inclusion promises to shed light on US cancer inequities, as fewer than 2% of cancer studies have been powered to consider race/ethnicity [14,15]. Given its diversity and large sample size, *All of Us* may have the statistical power to answer questions about the causes of cancer and drivers of disparities and identify opportunities for precision prevention.

Researchers currently have access to data from over 315K *All of Us* participants through the Researcher Workbench. Although the program does not target enrollment by health status, the sample to date includes a sufficient number of participants with a history of cancer, prevalent cancers, and incident cancers to enable systematic studies of cancer risk, outcomes, medication effects, and therapeutic approaches across environmental, social, genomic, and economic contexts. This demonstration project examines the distribution and characterization of cancer in *All of Us* and compares these numbers to expected national rates reported by the Surveillance, Epidemiology, and End Results (SEER) Program [16] and distribution in the US population.

## Materials and methods

### All of us research projects

The goals, recruitment methods and sites, and scientific rationale for *All of Us* have been described previously [17]. Demonstration projects were designed to establish the value of the cohort by describing the cohort and replicating previous findings for validation [18]. The work described here was proposed by Consortium members, reviewed and overseen by the program’s Science Committee, and was confirmed as meeting criteria for non-human subjects research by the *All of Us* Institutional Review Board. The initial release of data and tools used in this work was published in 2020 [18].

This work was performed using the *All of Us* Researcher Workbench, a cloud-based platform where approved researchers can access and analyze *All of Us* data. At the time of analysis, the *All of Us* data included survey responses, Electronic Health Records (EHR), and physical measurements (PM). These three types of data are collected either at an *All of Us* affiliated health care provider organization (HPO) or through a “direct-volunteer” mechanism. HPOs include regional medical centers, federally qualified health centers, and the Veterans Health Administration. HPOs recruit the majority of program participants–mainly persons affiliated with their center. The direct-volunteer route allows those who are not HPO patients to enroll online and visit a designated health clinic, blood bank, laboratory, or health care provider organization to have their PM collected. All three data types (survey, PM, and EHR) were mapped to the Observational Health and Medicines Outcomes Partnership (OMOP) common data model v 5.2 maintained by the Observational Health and Data Sciences Initiative (OHDSI) collaborative

To protect participant privacy, a series of data transformations were applied. These included data suppression of codes with a high risk of identification such as military status; generalization of categories, including age, sex at birth, gender identity, sexual orientation, and race; and date shifting by a random (less than one year) number of days, implemented consistently across each participant record. Documentation on privacy implementation and creation of the CDR is available in the *All of Us* Registered Tier CDR Data Dictionary [19]. The Researcher Workbench currently offers tools with a user interface (UI) built for selecting groups of participants (Cohort Builder), creating datasets for analysis (Dataset Builder), and Workspaces with Jupyter Notebooks (Notebooks) to analyze data. The Notebooks enable use of saved datasets and direct query using R and Python 3 programming languages.

### Study population

Participant-provided information for our analysis was derived from the surveys described above. The full text of these surveys is available in the Survey Explorer found in the *All of Us* Research Hub, a publicly available website designed to support researchers [20]. The *Basics* survey elicits demographic information including age, race/ethnicity, education, marital status, household income, and geography. The *Lifestyle* survey collects tobacco use data. *Personal Medical History* collects self-reported cancer history, including cancer type(s), life stage at diagnosis, and whether the participant is currently seeing a health care provider and/or receiving cancer treatment. The *Basics* and *Lifestyle* surveys are collected at baseline, whereas *Personal Medical History* is collected during retention efforts 3 months after enrollment.

Cancer diagnosis data were also derived from participant EHR. Diagnoses were determined using SNOMED CT codes and mapped to OMOP concept ID by the *All of Us* DRC. SNOMED CT codes for cancers and subtypes were combined to reflect the categories used for national reporting, including SEER and the North American Association of Central Cancer Registries (NACCR). EHR data also include procedures, medications, laboratory tests, and health care provider visits. We used the following cancers/cancer sites in our analysis: bladder (93689003), leukemia (93143009), non-Hodgkin’s lymphoma (118601006), myeloma (109989006), bone (93725000), brain (93727008), breast (372137005), cervix (372024009), colon (93761005), endocrine system (371983001), endometrium (10708511000119100), esophagus (371984007), eye (371986009), head/neck (372123001), kidney (93849006), lung (93880001), oral cavity (372001002), ovary (93934004), pancreas (372003004), prostate (93974005), rectum (93984006), stomach (372014001), and thyroid (94098005).

Categories of time from diagnosis were taken from the *Personal Medical History* survey, which asks: “About how old were you when you were first told you had this condition?” Response categories were child (0–11), adolescent (12–17), adult (18–64), older adult (65–74), and elderly (75+).

Time from diagnosis in the EHR was calculated as the current date minus the date of diagnosis, reported in years (mean, SD, and median).

Treatment type was reported for persons with a history of cancer from the EHR using the following SNOMED codes: surgery (1623, 11600, 11601, 11602, 11603, 11604, 11606, 11620, 11621, 11622, 11624, 11626, 11640, 11641, 11642, 11643, 11644, 11646, 17260, 17261, 17262, 17263, 17264, 17266, 17270, 17272, 17273, 17274, 17276, 17280, 17281, 17282, 17283, 17284, 17286, 370612006), radiotherapy (108290001), chemotherapy (38216008), immunotherapy (64644003), hormone therapy (10324, 72143, L02BB, L02BG), and stem cell transplant (41.04, 41.05, 41.06, 41.07, 41.08).

### National comparison

We compared the observed frequency of cancer reported in *All of Us* to National Cancer Institute’s SEER 18 Registries Database, November 2018 submission [21], to analyze cancer frequency overall and by site based on cases diagnosed in 2016 among residents of the areas included in the 18 registries covering ∼28% of the United States population. We reported the frequency of diagnosis in 2016 by assessing the limited duration 26-year cancer prevalence to determine the relative frequency and percent contribution of each cancer type to all cancers in the population by evaluating prevalence data representing the first invasive tumor site. Limited-Duration Prevalence represents the proportion of people alive on a certain day who had a diagnosis of the disease within the past x years (e.g. x = 5, 10 or 20 years). We chose the most recent year of diagnosis given the period for which *All of Us* has been conducting enrollment. Skin cancer (melanoma of the skin) was excluded from the “total cancer” calculation for SEER cancers and from the analysis since the *All of Us* survey data does not differentiate between melanoma and non-melanoma skin cancer. Invasive cancer was coded using the International Classification of Diseases for Oncology, third edition (ICD-O-3) [22].

### Data analysis

We generated descriptive statistics and prevalence for the most common cancers and used Chi-square tests to test the difference in the categorical distribution of data source types (survey data, EHR, and both) across the key demographic and lifestyle categories. The percent distribution of cancer types was calculated as the number of cases per site/total number of cancer cases in each respective dataset. Results are stratified by race/ethnicity and sex at birth to consider the demographic-specific distributions in cancer types. Cancer frequency was calculated using SEER*Stat 8.3.9 [23].

## Results

Table 1 shows the distribution of the baseline characteristics of all participants (N = 315,297), and by those with a cancer outcome as captured from the EHR (N = 203,813 participants with EHR; including N = 23,520 cancer cases), via self-report in the survey database (N = 89,261 completed *Personal Medical History* survey; including N = 13,298 cancer cases), and from participants with both survey and EHR data (N = 62,497 participants with both data types; including N = 7,123 cancer cases). *Personal Medical History* survey completion varies considerably, with older, female, and non-Hispanic Whites more likely to provide data than the population with available EHR (that more closely reflects the larger *All of Us* participant population). Differences across key demographic factors in data availability (survey data and/or EHR) are reflected in the distribution of cancer from the different data sources. Specifically, 84.8% of cancers from the *Personal Medical History* survey were reported by non-Hispanic Whites, 5.0% by Blacks, and 4.7% by Hispanics compared to 67.1%, 14.3%, and 12.2% respectively captured from the EHR. Non-Hispanic Whites are overwhelmingly represented among those with both self-report and EHR data (75.8%) compared to 51.5% representation in the overall *All of Us* study population. All p-values for the chi-square values comparing the distributions are <0.001 except the comparison of EMR versus total (which is 0.002).

**Table 1:** Comparison of characteristics of participants with cancer to the broader *All of Us* study population using self-reported survey data and electronic health record.

		Overall AoU Population		Participants with survey data	Cancer from survey	% of persons with history of cancer	Participants with EHR	Cancer from EHR	% of persons with history of cancer	Participants with survey and EHR data	Cancer from survey and EHR data	% of persons with history of cancer
		N	%	N	N	prevalence	N	N	prevalence	N	N	prevalence
Total		315,297		89,261	13,298	14.9%	203,813	23,520	11.5%	62,497	7,123	11.4%
Age	20–35	65,173	20.7%	14,919	370	2.5%	39,035	719	1.8%	9,506	150	1.6%
	35–50	71,695	22.8%	17,293	1,271	7.3%	44,621	2,374	5.3%	11,732	592	5.0%
	50–65	97,126	30.8%	25,999	3,924	15.1%	63,821	7,517	11.8%	18,500	2,028	11.0%
	65+	76,835	24.4%	30,679	7,731	25.2%	53,938	12,873	23.9%	22,572	4,353	19.3%
Gender	Female	191,114	60.6%	58,726	8,397	14.3%	124,735	13,994	11.2%	41,565	4,420	10.6%
	Male	119,750	38.0%	29,935	4,778	16.0%	76,162	9,183	12.1%	20,472	2,631	12.9%
	Other	3,866	1.2%	560	117	20.9%	490	55	11.2%	424	69	16.3%
Race/Ethnicity	NH White	162,330	51.5%	67,991	11,276	16.6%	105,678	15,776	14.9%	47,345	6,125	12.9%
	Black/AA	66,954	21.2%	6,746	669	9.9%	41,595	3,361	8.1%	4,971	328	6.6%
	Hispanic	59,283	18.8%	7,846	628	8.0%	40,217	2,870	7.1%	5,667	294	5.2%
	Asian	10,276	3.3%	3,000	207	6.9%	5,679	447	7.9%	1,903	107	5.6%
	Other	2,177	0.7%	495	54	10.9%	1,392	145	10.4%	354	29	8.2%
	>1 population	4,950	1.6%	1,527	152	10.0%	3,076	243	7.9%	1,001	61	6.1%
	None of these	3,343	1.1%	750	111	14.8%	2,178	211	9.7%	537	53	9.9%
	No answer	2,127	0.7%	657	162	24.7%	2,247	311	13.8%	521	~	~
Education	<HS	31,984	10.2%	1,922	163	8.5%	21,280	1,533	7.2%	1,632	70	4.3%
	HS/GED	64,006	20.3%	7,545	1,027	13.6%	42,601	3,607	8.5%	5,815	553	9.5%
	Some college	80,110	25.4%	20,221	3,134	15.5%	52,832	5,909	11.2%	14,566	1,584	10.9%
	College	131,462	41.7%	59,036	8,885	15.1%	82,174	12,028	14.6%	40,083	4,367	10.9%
	No answer	2,006	0.6%	424	71	16.7%	3,401	335	9.9%	324	~	~
Annual Household Income	<$35K	111,266	35.3%	17,864	2,233	12.5%	72,496	6,014	8.3%	12,929	1,032	8.0%
	$35-75K	55,902	17.7%	21,087	3,152	14.9%	35,819	4,598	12.8%	14,694	1,644	11.2%
	$75-150K	53,380	16.9%	25,574	3,929	15.4%	33,358	4,941	14.8%	17,436	2,160	12.4%
	$150K+	23,130	10.3%	16,584	2,607	15.7%	19,797	3,221	16.3%	11,253	1,466	13.0%
	No answer	29,672	13.2%	6,545	1,131	17.3%	29,000	3,443	11.9%	4,990	682	13.7%
Smoking Frequency	Not at all	66,334	53.3%	24,669	4,946	20.0%	46,168	8,097	17.5%	17,745	2,723	15.3%
	Some days	17,857	14.3%	1,990	221	11.1%	11,618	774	6.7%	1,435	98	6.8%
	Every day	38,129	30.6%	3,739	498	13.3%	24,609	1,375	5.6%	2,703	195	7.2%

*Skin cancer is excluded from the analysis as it is not differentiated as malignant/non-malignant/melanoma in AoU survey.

** Less than 20 cancers are excluded from analysis and indicated as ~.

Table 2 shows that *All of Us* participants’ EHR data indicate a history of breast cancer most frequently (N = 6,474; 27.5% of cases) followed by blood cancers (N = 4,841; 20.6%) and prostate cancer (N = 3,971; 16.9%). This mirrors the most common self-reported cancers (from the survey) for breast cancer (N = 4,062; 30.5%) and prostate cancer (N = 2,165; 16.3%) but not for blood cancer (N = 483; 9.9%). There are N = 2,499 individuals with breast cancer documented from both the survey and EHR data sources, followed by N = 1,304 individuals with prostate cancer cases, and followed by N = 657 blood cancer cases. Prevalence is broken down by cancer site showing the difference in contribution to disease burden by data source.

**Table 2:** The relative distribution and prevalence of cancer cases by type in the *All of Us* Research Program from self-reported survey data and electronic health record overall.

	EHR			Survey Data			EHR + Survey		
	N	% dist	prevalence	N	% dist	prevalence	N	% dist	prevalence
Population			203,813			89,261			62,497
Total Cancers	23,520	-	11.54%	13,298	-	14.90%	7,123	-	11.40%
Bladder	983	4.18%	0.48%	483	3.63%	0.54%	301	4.23%	0.48%
Blood	4,841	20.58%	2.38%	1,113	8.37%	1.25%	657	9.22%	1.05%
Bone	350	1.49%	0.17%	181	1.36%	0.20%	107	1.50%	0.17%
Brain	612	2.60%	0.30%	182	1.37%	0.20%	102	1.43%	0.16%
Breast	6,474	27.53%	3.18%	4,062	30.55%	4.55%	2,499	35.08%	4.00%
Cervix	576	2.45%	0.28%	869	6.53%	0.97%	172	2.41%	0.28%
Colon & Rectum	2,601	11.06%	1.28%	722	5.43%	0.81%	385	5.41%	0.62%
Endocrine System	1,887	8.02%	0.93%	129	0.97%	0.14%	63	0.88%	0.10%
Endometrium	1,364	5.80%	0.67%	459	3.45%	0.51%	212	2.98%	0.34%
Esophagus	230	0.98%	0.11%	110	0.83%	0.12%	60	0.84%	0.10%
Eye	123	0.52%	0.06%	66	0.50%	0.07%	28	0.39%	0.04%
Head & Neck	1,698	7.22%	0.83%	333	2.50%	0.37%	155	2.18%	0.25%
Kidney	1,266	5.38%	0.62%	487	3.66%	0.55%	313	4.39%	0.50%
Lung	1,081	4.60%	0.53%	463	3.48%	0.52%	283	3.97%	0.45%
Ovary	786	3.34%	0.39%	348	2.62%	0.39%	207	2.91%	0.33%
Pancreas	548	2.33%	0.27%	119	0.89%	0.13%	77	1.08%	0.12%
Prostate	3,971	16.88%	1.95%	2,165	16.28%	2.43%	1,304	18.31%	2.09%
Stomach	320	1.36%	0.16%	76	0.57%	0.09%	35	0.49%	0.06%
Thyroid	1,648	7.01%	0.81%	924	6.95%	1.04%	573	8.04%	0.92%

*Skin cancer is excluded from the analysis as it is not differentiated as malignant/non-malignant/melanoma in AoU survey.

Table 3 presents cancer type distribution from each data source by race and ethnicity, with N = 6,125 cancer cases detected in both data sources for non-Hispanic Whites compared to N = 328 cancer cases in African Americans and N = 294 cancer cases in Hispanics. Differences in the distribution of cancer types between survey data and EHR are observed by race/ethnicity (both within and between race/ethnicity, comparing non-Hispanic Whites, Blacks, and Hispanics (<0.001)). The prevalence of cancer subsequently varies by race/ethnicity in each data source as well as reported here.

**Table 3:** The relative distribution and prevalence of cancer cases by type in the *All of Us* Research Program from self-reported survey data and electronic medical record by race/ethnicity.

	NH-White								African American/Black									
	EHR			Survey data			EHR + Survey			EHR			Survey data			EHR + survey		
	N	% dist	prevalence	N	% dist	prevalence	N	% dist	prevalence	N	% dist	prevalence	N	% dist	prevalence	N	% dist	prevalence
Population			105,678			67,991			47,345			41,515			6,746			4,971
Total Cancer	15,776	-	14.9%	11,276	-	16.6%	6,125	-	12.9%	3,361	-	8.1%	669	-	9.9%	328	-	6.6%
Bladder	783	4.96%	0.74%	440	3.90%	0.65%	279	4.56%	0.59%	84	2.5%	0.20%	~	NA	~	~	NA	~
Blood	3,136	19.88%	2.97%	983	8.72%	1.45%	583	9.52%	1.23%	730	21.7%	1.76%	30	4.5%	0.44%	~	NA	~
Bone	229	1.45%	0.22%	142	1.26%	0.21%	86	1.40%	0.18%	41	1.2%	0.10%	~	NA	~	~	NA	~
Brain	398	2.52%	0.38%	151	1.34%	0.22%	89	1.45%	0.19%	92	2.7%	0.22%	~	NA	~	~	NA	~
Breast	4,382	27.78%	4.15%	3,422	30.35%	5.03%	2,124	34.68%	4.49%	875	26.0%	2.11%	210	31.4%	3.11%	125	38.1%	2.51%
Cervix	282	1.79%	0.27%	711	6.31%	1.05%	148	2.42%	0.31%	143	4.3%	0.34%	51	7.6%	0.76%	~	NA	~
Colon & Rectum	1,683	10.67%	1.59%	604	5.36%	0.89%	330	5.39%	0.70%	398	11.8%	0.96%	43	6.4%	0.64%	18	5.5%	~
Endocrine System	1,221	7.74%	1.16%	104	0.92%	0.15%	56	0.91%	0.12%	213	6.3%	0.51%	~	NA	~	~	NA	~
Endometrium	788	4.99%	0.75%	399	3.54%	0.59%	187	3.05%	0.39%	268	8.0%	0.65%	19	2.8%	~	~	NA	~
Esophagus	166	1.05%	0.16%	93	0.82%	0.14%	52	0.85%	0.11%	23	0.7%	0.06%	~	NA	~	~	NA	~
Eye	92	0.58%	0.09%	62	0.55%	0.09%	27	0.44%	0.06%	13	0.4%	~	~	NA	~	~	NA	~
Head & Neck	1,559	9.88%	1.48%	296	2.63%	0.44%	138	2.25%	0.29%	23	0.7%	0.06%	~	NA	~	~	NA	~
Kidney	817	5.18%	0.77%	399	3.54%	0.59%	258	4.21%	0.54%	212	6.3%	0.51%	31	4.6%	0.46%	22	6.7%	0.44%
Lung	700	4.44%	0.66%	389	3.45%	0.57%	244	3.98%	0.52%	219	6.5%	0.53%	25	3.7%	0.37%	~	NA	~
Other Site	NA	NA	~	1,499	13.29%	2.20%	601	9.81%	1.27%	~	NA	~	90	13.5%	1.33%	42	12.8%	0.84%
Ovary	501	3.18%	0.47%	284	2.52%	0.42%	170	2.78%	0.36%	103	3.1%	0.25%	25	3.7%	0.37%	~	NA	~
Pancreas	346	2.19%	0.33%	94	0.83%	0.14%	61	1.00%	0.13%	76	2.3%	0.18%	~	NA	~	~	NA	~
Prostate	2,837	17.98%	2.68%	1,915	16.98%	2.82%	1,155	18.86%	2.44%	605	18.0%	1.46%	114	17.0%	1.69%	60	18.3%	1.21%
Stomach	174	1.10%	0.16%	59	0.52%	0.09%	29	0.47%	0.06%	51	1.5%	0.12%	~	NA	~	~	NA	~
Thyroid	1,068	6.77%	1.01%	798	7.08%	1.17%	493	8.05%	1.04%	182	5.4%	0.44%	33	4.9%	0.00%	15	4.6%	~

	Hispanic								
	EHR			Survey			EHR + Survey		
	N	% dist	prevalence	N	% dist	prevalence	N	% dist	prevalence
Population			40,217			7,846			5,667
Total Cancer	2,870	-	7.1%	628	-	8.0%	294	-	5.19%
Bladder	69	2.4%	0.17%	~	NA	~	~	NA	~
Blood	663	23.1%	1.65%	42	6.7%	0.5%	27	9.2%	0.48%
Bone	51	1.8%	0.13%	~	NA	~	~	NA	~
Brain	83	2.9%	0.21%	~	NA	~	~	NA	~
Breast	786	27.4%	1.95%	191	30.4%	2.4%	111	37.8%	1.96%
Cervix	120	4.2%	0.30%	70	11.1%	0.9%	~	NA	~
Colon & Rectum	377	13.1%	0.94%	39	6.2%	0.5%	23	7.8%	~
Endocrine System	314	10.9%	0.78%	~	NA	~	~	NA	~
Endometrium	225	7.8%	0.56%	23	3.7%	0.29%	~	NA	~
Esophagus	22	0.8%	0.05%	~	NA	~	~	NA	~
Eye	~	NA	~	~	NA	~	~	NA	~
Head & Neck	53	1.8%	0.13%	~	NA	~	~	NA	~
Kidney	172	6.0%	0.43%	35	5.6%	0.4%	~	NA	~
Lung	81	2.8%	0.20%	~	NA	~	~	NA	~
Other Site	NA	NA	~	115	18.3%	1.5%	36	12.2%	0.64%
Ovary	114	4.0%	0.28%	20	3.2%	0.3%	~	NA	~
Pancreas	90	3.1%	0.22%	~	NA	~	~	NA	~
Prostate	295	10.3%	0.73%	45	7.2%	0.6%	25	8.5%	0.44%
Stomach	65	2.3%	0.16%	~	NA	~~	~	NA	~
Thyroid	280	9.8%	0.70%	70	11.1%	0.9%	35	11.9%	0.62%

*Skin cancer is excluded from the analysis as it is not differentiated as malignant/non-malignant/melanoma in AoU survey.

** Less than 20 cancers are excluded from analysis and indicated as ~.

Table 4 compares the distribution of cancer sites from *All of Us* survey data and EHR to the expected distribution nationally, based on recent SEER reports of the 26-year limited duration prevalence in 2018. The most common cancer types in SEER (based on contribution to total cancers) are breast cancer (19.9%), prostate cancer (17.6%), blood cancers (11.4%), and colorectal cancers (8.4%). The percent contribution to the cancer burden nationally (as illustrated by SEER data) from each cancer site differs significantly from the EHR site distribution (p<0.001) and the self-reported distribution (p<0.001). As expected, the percent of persons enrolled into All of Us largely from medical centers have a higher proportion of prevalent cancer (11.54% in EHR and 14.90% in survey) than in the US population reported by SEER (4.43%).

**Table 4:** Comparison of relative distribution and prevalence of cancer cases by type in the *All of Us* Research Program to SEER’s 26-year limited duration prevalence.

	EHR			Survey Data			EHR + Survey			SEER 26-year prevalence		
	N	% dist	prevalence	N	% dist	prevalence	N	% dist	prevalence	N	% dist	prevalence
Population			203,813			89,261			62,497			325,836,757
Total Cancers	23,520	-	11.54%	13,298	-	14.90%	7,123	-	11.40%	14,419,319		4.43%
Bladder	983	4.18%	0.48%	483	3.63%	0.54%	301	4.23%	0.48%	555,999	3.86%	0.17%
Blood	4,841	20.58%	2.38%	1,113	8.37%	1.25%	657	9.22%	1.05%	1,343,512	9.32%	0.41%
Bone	350	1.49%	0.17%	181	1.36%	0.20%	107	1.50%	0.17%	33,086	0.23%	0.01%
Brain	612	2.60%	0.30%	182	1.37%	0.20%	102	1.43%	0.16%	129,633	0.90%	0.04%
Breast	6,474	27.53%	3.18%	4,062	30.55%	4.55%	2,499	35.08%	4.00%	3,096,156	21.47%	0.95%
Cervix	576	2.45%	0.28%	869	6.53%	0.97%	172	2.41%	0.28%	182,868	1.27%	0.06%
Colon & Rectum	2,601	11.06%	1.28%	722	5.43%	0.81%	385	5.41%	0.62%	1,134,250	7.87%	0.35%
Endocrine System	1,887	8.02%	0.93%	129	0.97%	0.14%	63	0.88%	0.10%	70,825	0.49%	0.02%
Endometrium	1,364	5.80%	0.67%	459	3.45%	0.51%	212	2.98%	0.34%	632,326	4.39%	0.19%
Esophagus	230	0.98%	0.11%	110	0.83%	0.12%	60	0.84%	0.10%	21,960	0.15%	0.01%
Eye	123	0.52%	0.06%	66	0.50%	0.07%	28	0.39%	0.04%	~	~	~
Head & Neck	1,698	7.22%	0.83%	333	2.50%	0.37%	155	2.18%	0.25%	396,937	2.75%	0.12%
Kidney	1,266	5.38%	0.62%	487	3.66%	0.55%	313	4.39%	0.50%	451,550	3.13%	0.14%
Lung	1,081	4.60%	0.53%	463	3.48%	0.52%	283	3.97%	0.45%	423,209	2.94%	0.13%
Ovary	786	3.34%	0.39%	348	2.62%	0.39%	207	2.91%	0.33%	167,758	1.16%	0.05%
Pancreas	548	2.33%	0.27%	119	0.89%	0.13%	77	1.08%	0.12%	65,973	0.46%	0.02%
Prostate	3,971	16.88%	1.95%	2,165	16.28%	2.43%	1,304	18.31%	2.09%	3,017,103	20.92%	0.93%
Stomach	320	1.36%	0.16%	76	0.57%	0.09%	35	0.49%	0.06%	96,886	0.67%	0.03%
Thyroid	1,648	7.01%	0.81%	924	6.95%	1.04%	573	8.04%	0.92%	660,323	4.58%	0.20%

*Skin cancer is excluded from the analysis as it is not differentiated as malignant/non-malignant/melanoma in AoU survey.

* SEER data is based on 5-year prevalence frequency counts of 1^st^ invasive tumor.

Table 5 presents a description of the time from cancer diagnosis as reported in the EHR and survey database. The cancer with the shortest time from diagnosis in the EHR is lung cancer (mean = 5.85 years; SD = 4.46), and the longest time from diagnosis is for head and neck cancer (mean = 11.75 years; SD = 6.59). Across all cancer types, the most common period of diagnosis was adult, followed by older adult.

**Table 5:** Time from diagnosis of cancer cases and approximate age at diagnosis by type in the *All of Us* Research Program.

	AoU EHR			N with timing of diagnosis	Child		Adolescent		Adult			Older Adult	Elderly	
	Time between cancer diagnosis and enrollment, years													
Cancer Site	Mean	SD	Median	N	N	%	N	%	N	%	N	%	N	%
Bladder	7.97	6	6.35	478	~	NA	~	NA	249	50.00%	187	38.10%	39	10.80%
Blood	7.19	5.04	5.47	1,105	26	2.50%	24	0.90%	754	69.10%	246	21.90%	55	5.30%
Bone	8.62	6.57	6.59	180	~	NA	~	NA	114	427.10%	40	135.70%	~	NA
Brain	8.83	6.68	6.78	181	9	4.90%	~	NA	140	76.90%	~	NA	~	NA
Breast	8.07	5.4	6.91	4,039	~	NA	~	NA	3,328	81.90%	626	15.40%	83	2.00%
Cervix	8.42	6.16	6.59	863	~	NA	23	2.60%	813	93.60%	21	2.40%	~	NA
Colon & Rectum	7.94	5.41	6.81	717	~	NA	~	NA	535	74.10%	152	21.10%	27	3.70%
Endocrine System	8.19	5.65	6.81	128	~	NA	~	NA	95	73.60%	23	17.80%	~	NA
Endometrium	7.6	5.66	5.84	457	~	NA	~	NA	383	83.40%	66	14.40%	~	NA
Esophagus	6.55	4.92	5.17	110	~	NA	~	NA	64	58.20%	42	38.20%	~	NA
Eye	7.09	5.22	5.73	66	~	NA	~	NA	44	66.70%	~	NA	~	NA
Head & Neck	11.75	6.59	11.2	330	~	NA	~	NA	222	66.70%	89	26.70%	~	NA
Kidney	7.19	5.29	5.81	485	~	NA	~	NA	340	69.80%	118	24.20%	~	NA
Lung	5.85	4.46	4.38	459	~	NA	~	NA	243	52.50%	172	37.10%	42	9.10%
Ovary	8.24	5.99	6.34	347	~	NA	~	NA	296	85.10%	41	11.80%	~	NA
Pancreas	6.48	5.1	4.57	117	~	NA	~	NA	71	59.70%	34	28.60%	~	NA
Prostate	7.95	5.44	6.57	2,157	~	NA	~	NA	1,254	57.90%	807	37.30%	96	4.40%
Stomach	6.83	5.14	4.88	76	~	NA	~	NA	59	77.60%	~	NA	~	NA
Thyroid	8.17	5.67	6.78	921	~	NA	~	NA	789	85.40%	100	10.80%	~	NA

*Skin cancer is excluded from the analysis as it is not differentiated as malignant/non-malignant/melanoma in AoU survey.

** Less than 20 cancers are excluded from analysis and indicated as ~.

Table 6 presents treatment types for cancer overall and by site. The most common treatment from the EHR is radiation (N = 7,422; 31.56%), followed by surgery (N = 5,975; 25.54%), hormone therapy (N = 3,962; 16.84%), chemotherapy (N = 842; 3.58%), immunotherapy (N = 470; 1.2%), and stem cell transplant (N = 127; 0.54%). Treatment type utilization varied by cancer site.

**Table 6:** Cancer treatment type overall and by site in the *All of Us* Research Program.

		Surgery		Radiation		Chemotherapy		Immunotherapy		Hormone Therapy		Stem Cell Transplant	
Cancer Site	Total AoU EHR Cases	N	%	N	%	N	%	N	%	N	%	N	%
All Sites	23,520	5,975	25.4%	7,422	31.56%	842	3.58%	470	1.2%	3,962	16.84%	127	0.54%
Bladder	983	185	18.82%	130	13.22%	24	2.44%	~	~	48	4.88%	~	~
Blood	4,841	666	13.76%	724	14.96%	322	6.65%	192	3.97%	210	4.34%	96	1.98%
Bone	350	98	28%	128	36.57%	~	~	~	~	39	11.14%	~	~
Brain	612	192	31.37%	235	38.4%	~	~	~	~	29	4.74%	~	~
Breast	6,474	1,075	16.6%	1,898	29.32%	132	2.04%	97	1.5%	2,445	37.77%	~	~
Cervix	576	74	12.85%	132	22.92%	~	~	~	~	25	4.34%	~	~
Colon & Rectum	2,601	455	17.49%	478	18.38%	83	3.19%	30	1.15%	127	4.88%	~	~
Endocrine System	1,887	338	17.91%	502	26.6%	23	1.22%	~	~	95	5.03%	~	~
Endometrium	1,364	194	14.22%	332	24.34%	23	1.68%	~	~	90	6.6%	~	~
Esophagus	230	30	13.04%	79	34.35%	~	~	~	~	~	~	~	~
Eye	123	23	18.7%	26	21.14%	~	~	~	~	~	~	~	~
Head & Neck	1,698	916	53.95%	241	14.19%	~	~	~	~	71	4.18%	~	~
Kidney	1,266	206	16.27%	175	13.82%	~	~	~	~	62	4.9%	~	~
Lung	1,081	180	16.65%	343	31.73%	26	2.41%	~	~	61	5.64%	~	~
Ovary	786	119	15.14%	124	15.78%	31	3.94%	~	~	62	7.89%	~	~
Pancreas	548	78	14.23%	130	23.72%	~	~	~	~	29	5.29%	~	~
Prostate	3,971	612	15.41%	879	22.14%	29	0.73%	~	~	421	10.6%	~	~
Stomach	320	57	17.81%	76	23.75%	~	~	~	~	~	~	~	~
Thyroid	1,648	286	17.35%	449	27.25%	~	3.58%	~	~	77	4.67%	~	~

*Skin cancer is excluded from the analysis as it is not differentiated as malignant/non-malignant/melanoma in AoU survey.

** Less than 20 cases are excluded from analysis and indicated as ~.

## Conclusions

In this preliminary analysis of data from the *All of Us* Research Program, we report that the first 315K+ persons comprise a diverse population with a large number of prevalent cancer cases. As the goal of this effort is to inform studies on a variety of health conditions, including cancer, and to delineate information on risk factors and treatments, an early evaluation of cancers represented in the study population is warranted. Our findings have some key implications for cancer prevention, control, treatment, and outcomes research in the *All of Us* study population.

Our most notable finding is simple: although a diverse cohort is being enrolled, self-reported cancers are not being ascertained as frequently through the survey modules among underrepresented participants. As validation of diagnosis from EHR using manual verification or self-report is the gold standard to ensure accurate classification and minimize measurement error, the difference in valid case ascertainment by key factors like race is relevant for *All of Us* cancer research. The drop in cancer data detected from the survey or validated with survey data is associated with racial/ethnic differences in longitudinal retention. Although surveys are completed by a relatively older population, age doesn’t appear to be a key factor influencing differences in data collection. History of cancer is collected through a survey completed at least 90 days after enrollment in *All of Us*, with an overall medical history survey completion rate among underrepresented participants of 22% across the program compared to 42% in non-UBR participants. Some factors noted in the literature previously [24] that could be of relevance for differences in retention by race/ethnicity include language, literacy, cultural appropriateness, flexibility, ongoing incentives, communication, and of particular growing importance with increasingly electronic survey data collection is the digital divide. This has research implications for the cancer history data collected at follow-up as well as other key risk factor information including health care utilization, personal medical history, and family history. Our investigation shows that the impact of these factors on cancer disparities will be underreported even if cancer history can be obtained from the EHR of most underrepresented participants. *All of Us* leadership has changed survey module timeline and made *Personal Medical History* available at baseline, addressing some of the limitations noted here for prospective enrollees.

Furthermore, the difference in cancer ascertainment between survey modules and EHR modalities in underrepresented participants highlights the importance of technologies to integrate the medical records of direct volunteers. Sync for Science for obtaining EHRs from direct volunteers or other non-digital methods of collecting survey data could offer utility beyond the ability to confer medical record information for direct volunteers, as there are implications for inclusion and equity in the investigation of all diseases, including cancer.

The distribution of cancer sites between the two data sources when compared to SEER national statistics is impacted by exclusion of skin cancer from the *All of Us* cancer analyses. Skin cancer cases account for approximately half of the total cases reported in the survey data. These cases likely include both malignant and non-malignant skin cancers, which would introduce significantly different relative proportions of other cancers if included in the analysis. As restriction to malignant cases was not possible, we excluded all skin cancer cases from analysis.

Another point to consider is the grouping of blood cancers. Because the survey module asks about blood cancers generically, it is impossible to differentiate between myeloma, lymphoma, and leukemia in survey responses. This distinction can be deciphered from the EHR when available. The ability to distinguish these types will be crucial to many cancer researchers.

We further report on the time from diagnosis and the life stage to consider opportunities to collect incident cases or investigate hypotheses for more recent diagnoses. The utility of the life stage questions in etiology or outcomes research is unclear, as the groups (age ranges (child (0–11); adolescent (12–17); adult (18–64); older adult (65–74) and elderly (75+)) are quite broad in the survey. A more refined or consistent metric, such as date of cancer diagnosis, would aid investigation of various cancer-related hypotheses (such as being able to stratify by pre and post menopausal breast cancer. Presenting this data side-by-side highlights how distinct these metrics of diagnosis timing really are.

The *All of Us* Research Program is set to become one of the largest scientific efforts in U.S. history, and its emphasis on inclusion presents key opportunities to advance precision health and medicine and address disparities in research [25]. Despite the limitations noted in this report, this unprecedented depth of inclusion will confer an important resource for cancer research. *All of Us* was conceived to support studies of disease outcomes, medication effects, and other therapeutic approaches across various environmental, social, genomic, and economic contexts [26]. The scale and scope of its current cancer data will support extensive investigation of cancer-related hypotheses and enhance the pace of discovery and generalizability. The cohort’s expansion to 1 million participants will create further opportunities. Furthermore, feedback from demonstration projects such as this one will directly inform edits to existing surveys and development of reassessment modules.

In summary, the *All of Us* Research Program has collected significant cancer data from its first 315K participants. This preliminary investigation notes the most common cancers that will confer sufficient study power for research, especially once whole genome data is available for all participants. Considering our findings, the program might consider the implications of lower retention through survey completion among underrepresented participants on the resource’s utility for research on cancer and other diseases.
